# Activation of CD4 and CD8 T cell receptors and regulatory T cells in response to human proteins

**DOI:** 10.7717/peerj.4462

**Published:** 2018-03-09

**Authors:** Borros M. Arneth

**Affiliations:** Institute of Laboratory Medicine and Pathobiochemistry, Molecular Diagnostics, University Hospital of the Universities of Giessen and Marburg UKGM, Justus Liebig University Giessen, Giessen, Hessen, Germany

**Keywords:** Regulatory T cells, CD4+ T helper cells, Lymphocytes, CD8+ cytotoxic T cells, Interferon-gamma

## Abstract

This study assessed in detail the influence of four different human proteins on the activation of CD4+ and CD8+ T lymphocytes and on the formation of regulatory T cells. Human whole-blood samples were incubated with four different human proteins. The effects of these proteins on the downstream immune-system response, on the expression of extracellular activation markers on and intracellular cytokines in T lymphocytes, and on the number of regulatory T cells (T-reg cells) were investigated via flow cytometry. Incubation with β-actin or glyceraldehyde 3-phosphate dehydrogenase (GAPDH), which are cytoplasmic proteins, increased the expression of both extracellular activation markers (CD69 and HLA-DR) and intracellular cytokines but did not significantly affect the number of T-reg cells. In contrast, incubation with human albumin or insulin, which are serum proteins, reduced both extracellular activation markers and intracellular cytokine expression and subsequently increased the number of T-reg cells. These findings may help to explain the etiological basis of autoimmune diseases.

## Introduction

The stability and plasticity of various T lymphocyte subgroups parallel their various physiological functions and suggest selection mechanisms within the context of T cell maturation (Shah & Zúñiga-Pflücker, 2014). The various T helper subsets (i.e., Th1, Th2, and Th17) and regulatory T cells (T-reg cells) are interconvertible. Over time, T helper cells can differentiate into other cell types of this group via the activation of specific transcription factors. Indeed, the T cell receptor (TCR) spectrum of various T helper subsets is plastic, and such plasticity in terms of TCR spectra and of the antigens recognized results in various subgroups of T helper cells. Notably, the expression of CD4 and/or CD8 co-receptors—and thus differentiation into one of these two cell types—is fixed once it has occurred (Shah & Zúñiga-Pflücker, 2014).

The respective TCR spectra of T cells are fixed into a CD4+ TCR spectrum and/or a CD8+ TCR spectrum through stable differentiation into CD4+ and/or CD8+ T cells. This differentiation occurs at an extremely early stage during development through positive and negative selection of the respective T cells during thymic T cell development (Shah & Zúñiga-Pflücker, 2014). Although the plasticity of T helper cell subgroups allows increased variability in T cell populations later in life, fixation of CD4 and CD8 TCR spectra (Shah & Zúñiga-Pflücker, 2014) leads to permanent changes throughout the lifetime of an organism.

Several authors have described immunological memory as nothing more than “leftover” cells from former immunizations (Zinkernagel et al., 1996; Zinkernagel, 2003; Zinkernagel & Hengartner, 2006). Therefore, improved knowledge of CD4+ and CD8+ T cell activation could lead to a detailed understanding of both immunological memory as a “leftovers” of specific activated CD4+ and CD8+ T cells and the immune reactions that occur later in life.

### Core idea/core hypothesis

The present study hypothesized that self-molecules can be recognized as foreign when at the wrong location. To confirm or disprove this hypothesis, whole blood from healthy subjects was incubated with various human proteins, and the response regarding CD4+ and CD8+ T lymphocyte activation was examined.

To investigate the detailed effects of human proteins on CD4 + and CD8+ T lymphocytes and T-reg cells, whole-blood samples from healthy volunteers (*N* = 20) were incubated with four different human proteins (GAPDH, β-actin, insulin, and albumin).

These proteins were selected because they are prominent intracellular (actin and GAPDH) and extracellular (albumin, insulin), which were also purely available.

Then, the degree of the resulting T cell activation was quantified via flow-cytometric measurements of surface markers: CD69, which is an early T cell activation marker; HLA-DR, which is a late T cell activation marker, CD4, which is a T helper cell marker (CD4+ T cells); CD8, which is a cytotoxic T cell marker (CD8+ T cells); and CD3, which is predominantly located on T cells. Intracellular cytokine expression levels were also examined.

### The development of autoimmune diseases and the role of regulatory T cells

The hypotheses accepted to date assumes that autoimmune diseases are acquired through innate susceptibility, including genetic predisposition, in combination with external influences. Genetic predisposition is primarily because different individuals express different MHC molecule variants on the surfaces of their cells. Some MHC variants present more components that are similar to the body’s own structures, triggering an autoreactive immune response. Such genetic factors, in conjunction with stress-causing environmental factors such as viruses, other infections, and pregnancy, can result in an autoimmune disease. Another theory is the hygiene hypothesis, which holds that the development of immune diseases may be promoted by too little confrontation of the immune system with bacteria in the environment. Overall, women are more often affected by autoimmune diseases.

Furthermore, T cell selection during maturation in the thymus is considered important. Autoreactive T cells may dominate if they are not eliminated in the appropriate manner. In addition, cross-reactivity of bacterial and/or viral protein fragments and self-protein fragments might be important in the development of autoimmune diseases. Lastly, regulatory T cells (T-reg cells) are important in controlling effector T cells and preventing the development of autoimmune diseases. The mechanisms by which T-reg cells control effector T cells are complex.

## Methods

### Ethics approval and consent to participate

The institutional review board of the University of Mainz (Mainz, Germany) approved this research (ukm-2005-2742834). All participants gave written informed consent to participate in the study, and the consent procedure was approved by the institutional review board of the University of Mainz. All research was carried out in compliance with the Helsinki Declaration. All methods were performed in accordance with the relevant guidelines and regulations.

### Subjects

Whole-blood samples treated with ethylenediaminetetraacetic acid (EDTA) were collected from healthy volunteers (*N* = 20). The samples were incubated with and without various human proteins and analyzed by flow cytometry.

#### Flow cytometry

The samples were analyzed using a FACSCalibur (Becton Dickinson, San Diego, CA, USA) flow cytometer. An antibody mixture containing antibodies against CD4, CD8, CD3, and CD69 and/or antibodies against CD4, CD8, CD3, and HLA-DR (Becton Dickinson; San Diego, CA, USA) was added to 50 µl of each sample. The anti-CD69 antibody and/or the anti-HLA-DR antibody were conjugated with R-phycoerythrin (R-PE), the most sensitive fluorochrome among those available for staining. The antibodies specific to CD8, CD3, and CD4 were conjugated with fluorescein isothiocyanate (FITC), peridinin chlorophyll protein (PerCP), and allophycocyanin (APC), respectively.

FITC, PerCP, APC, and PE fluorescence levels were measured and graphed. The cells were initially gated based on the CD3 PerCP signal and side scatter. The CD3+ population was considered the T lymphocyte population. In total, 30,000 events in the T lymphocyte gate were recorded per sample.

#### Lymphocyte activation

The whole-blood sample from each individual was divided into 7 1-ml aliquots. The samples were either treated or untreated, as follows: Tube 1, concanavalin A as positive control (Sigma-Aldrich, Bremen, Germany); Tube 2, untreated sample as negative control; Tube 3, human albumin (Fresenius, Bad Homburg, Germany); Tube 4, human β-actin (Abcam; Cambridge, UK); Tube 5, human glyceraldehyde 3-phosphate dehydrogenase (GAPDH; Sigma-Aldrich, Bremen, Germany); Tube 6, human insulin (Eli Lilly; Bad Homburg, Germany); and Tube 7, untreated sample as negative control. All proteins were used at the same two concentrations (low: 2 µg/ml and high: 2 mg/ml), which were confirmed using the Bradford assay. For incubations at the 2-mg/ml concentration, the proteins were pre-incubated with a BioTrek transfection reagent (Stratagene, La Jolla, CA, USA). For intracellular cytokine assays (IL-2, IFg) and for HLA-DR activation, anti-CD28 and CD49d were used to co-stimulate T cells.

#### Incubation period

For the CD69 assay whole-blood samples with or without added proteins were placed in an incubator at 37 °C (98.6 °F) for 14 h (overnight). The samples were gently mixed approximately once every 2 h. Flow-cytometric analyses were performed the following day.

For the HLA-DR assay lymphocyte cultures were incubated at 37 °C (98.6 °F) for 72 h.

#### Protein purity

All experiments were performed using proteins isolated from human plasma, except for insulin, which was a recombinant protein identical to human insulin. All proteins were purified using the Protein Purification Kit from Norgen (BioCat, Thorold, ON, Canada) based on a spin column chromatography. Limulus amebocyte lysate (LAL) was used to ensure that no additives and/or pyrogens were included in the protein solutions (<0.1 IU/mg pyrogen by LAL).

#### Erythrocyte lysis

Erythrocyte lysis was performed prior to culturing for the HLA-DR, the intracellular interferon gamma activation, the interleukin-2 experiments as well as for the Treg cell analysis (pleases sees below). On the other hand, for the lymphocyte activation experiments by overnight incubation and CD69 measurement (overnight incubation with antigens, please see above) whole blood samples were used. In this cases the erythrocyte lyses was done after overnight incubation (after 14 h). Erythrocytes were lysed for 20 min with 450 µl of BD lysis buffer (1×) according to the manufacturer’s instructions (Becton Dickinson, San Diego, CA, USA). For CD69 measurements the samples were then analyzed by flow cytometry.

For culturing lymphocytes cells were isolated and cleaned by using anti- human CD3 MACS microbeads (MACS; Miltenyi Biotec, Bergisch Gladbach, Germany) prior to taking lymphocytes into culture. Culture medium was Dulbecco modified eagle medium (DMEM, Gibco, USA).

#### Live/dead-cell assays

For all experiments, samples (tubes) were examined for live and dead cells using a Live/Dead Viability Cytotoxicity Kit (Invitrogen MP 03224; Invitrogen, Carlsbad, CA, USA) for mammalian cells from Molecular Probes Invitrogen. Only experiments with 95% or more cells alive have been included into the evaluation.

#### Analysis of the intracellular activation markers interferon-gamma (IFN-γ) and interleukin-2 (IL-2)

Flow-cytometric analysis of the intracellular activation marker IFN-γ and IL-2 were conducted using BD FastImmune CD4 and/or CD8 Intracellular Cytokine Staining Kits.

1. The BD FastImmune CD4 Intracellular Cytokine Three-Color Kit contained the following:
  Anti-Hu- IFγ Kit (BD Cat. No. 340970) and/or Anti-Hu-IL-2 FITC
  Anti-Hu- IFγ FITC/CD69 PE/CD4 PerCP-Cy5.5 and/or
  Anti-Hu- IL2 FITC/CD69 PE/CD4 PerCP-Cy5.5
  Isotype controls IgG2a FITC/IgG1 PE/CD4 PerCP-Cy5.5 Activation and processing solutions.
2. The BD FastImmune CD8 Intracellular Cytokine Four-Color Kit contained the following:
  Anti-Hu- IFγ Kit (BD Cat. No. 346049) and/or Anti-Hu-IL-2 FITC
  Anti-Hu- IFγ FITC/CD69 PE/CD8 PerCP-Cy5.5/CD3 APC and/or
  Anti-Hu-IL-2 FITC/CD69 PE/CD8 PerCP-Cy5.5/CD3 APC
  Isotype controls IgG2a FITC/IgG1 PE/CD8 PerCP-Cy5.5/CD3 APC Activation and processing solutions.

Staining and flow-cytometric measurements were performed after the lymphocytes were incubated with and without the human proteins and co-stimulatory antibodies (CD28 and CD49d), as described above. To inhibit intracellular transport, Brefeldin A (BFA) was added during the last 4 h of activation. Measurements were performed according to the manufacturer’s instructions (FastImmune CD4/CD8 Intracellular Cytokine Kits; Becton Dickinson, San Diego, CA, USA). Cells were lysed using BD lysing solution (BD Cat. No. 347691), washed, and then permeabilized using BD FACS permeabilizing solution (BD Cat. No. 340973; Becton Dickinson, San Diego, CA, USA). Intracellular IFN-γ and/or IL-2 staining was performed using the FastImmune CD4 and/or CD8 Intracellular IFN-γ Kit (BD Cat. Nos. 340970 and 346049; Becton Dickinson, San Diego, CA, USA) and/or the IL-2 Kit.

#### T-reg cell analysis

Treg cell induction was initiated by addition of TGFβ and IL2 (Sigma Aldrich, St. Louis, MO, USA) to cell culture. Flow-cytometric analysis of T-reg cells was performed after staining CD25+, CD4+, and foxp3 T-reg cells using a BD Anti-human FoxP3 Staining Kit (BD Pharmingen, San Diego, CA, USA) containing FoxP3-PE, CD25-APC, and CD4-FITC. Staining and flow-cytometric measurements were performed after the samples were incubated with and without proteins, as described above, for a longer period. Flow cytometry was performed according to the manufacturer’s instructions.

### Statistical analysis

All experiments were performed three times per subject (*N* = 20) using whole-blood samples collected from each. The results of the experiments did not vary by more than 5% between two experiments for each subject, and the median was used for further statistical analysis.

Data were analyzed using the SPSS-16 software program (SPSS Inc., Chicago, IL, USA). Descriptive statistics (mean standard deviation [SD] were determined (normally distributed variables, tested with the Shapiro–Wilk test) variables. Baseline and clinical characteristics were compared by one-way analysis of variance (ANOVA). Student’s *t*-test was used for comparisons between groups. A *p*-value of <0.05 was considered statistically significant.

First, the percentage of activated cells was determined by dividing the number of double-positive cells (CD4- and CD69-positive cells and/or CD8- and CD69-positive cells) and/or CD4- and HLA-DR-positive cells and/or CD8- and HLA-DR-positive by that of the respective single-positive cells (CD4-positive and/or CD8-positive cells). For the statistical analysis, the experimental results were assigned to the following groups: Tube/Group 1, samples treated with concanavalin A as positive control; Tube/Group 2, untreated control samples as negative control (background); Tube/Group 3, samples treated with human albumin; Tube/Group 4, samples treated with human β-actin; Tube/Group 5, samples treated with human GAPDH; Tube/Group 6, samples treated with human insulin; and Tube/Group 7, untreated samples as second negative control samples (background).

ANOVA tests with Bonferroni correction (SPSS 16) were performed on the results for these groups, and several Student t-tests were performed to compare samples in each of these groups with the untreated samples in Tubes/Samples 2 and 7. The ANOVA showed that Tube/Group 1 and Tubes/Groups 3–6 differed from Tube/Group 2. The groups 2 and 7 did not differ. Student’s *t*-test demonstrated that Tubes/Groups 4, 5, and 6 differed significantly from Tube/Group 2. Because of this finding, the results of the experiments were documented as differences compared with the respective untreated samples (Tube/Group 2 samples).

Overall, the results showed that treatment with β-actin or GAPDH produced statistically significant results for both the CD4 and CD8 quotients (q4, q8). Conversely, no T cell activation was observed after treatment with low concentrations of albumin or insulin. After the samples were incubated overnight with albumin or insulin, a smaller percentage of activated T cells was observed compared with samples incubated with equal amounts of β-actin or GAPDH.

To enable a more detailed analysis, the samples were split into two subgroups: subgroup A, containing samples treated with a low (2 µg/ml) concentration of protein; and subgroup B, containing samples treated with a high (2 mg/ml) concentration of protein. The protein concentrations were confirmed by Bradford measurements at 595 nm.

The results of the present study detected an abundance of activated T lymphocytes, which were reported as percentages of CD4+ and CD8+ T lymphocyte subpopulations. The actual numbers of cells were considered the actual recorded cell distributions, and the cell distributions in quadrants (single-positive, double-positive, or double-negative) had almost no influence on the percentages recorded (the q quotients). Furthermore, comparison of the differences between treated and untreated samples ensured that the results were independent of the history of the subjects from whom the blood samples were obtained. Nonetheless, only healthy subjects were selected for these experiments.

## Results

**Figure 1:**
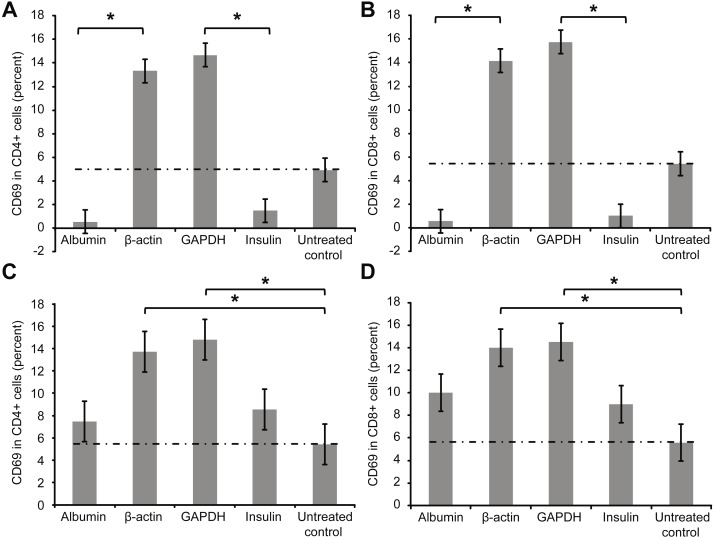
Results of extracellular staining for activated T cell surface marker CD69 on CD4+ (A) and CD8+ T cells (B). The percentage of activated cells decreased among both CD4+ and CD8+ T cells incubated overnight with albumin or insulin, indicating their inhibition. In contrast, the percentage of activated cells increased among CD4+ and CD8+ T cells incubated with an equal concentration of β-actin or GAPDH (*statistically significant difference, *p* < 0.01). Increase in the concentration (2 mg/ml) of the protein (C and D) slightly increased the percentages of activated CD69+ CD4+ and CD69 + CD8+ double-positive T cells.

Figure 1 presents the extracellular staining results for CD69, a surface marker for activated T cells. Significantly smaller percentages of activated T cells were observed after whole-blood samples were incubated overnight with low concentrations (2 µg/ml) of albumin or insulin, indicating that T cell inhibition occurred in these experiments. In contrast, the percentage of activated T cells increased after overnight incubation with 2 µg/ml of β-actin or GAPDH. Notably, incubating whole blood with a high concentration of albumin or insulin (2 mg/ml) led to a slight increase in the percentages of activated T cells (Figs. 1C and 1D). The albumin incubation experiments were also repeated using even higher concentrations of albumin (5 and 10 mg/ml), with no relevant differences in the results compared with those with 2 mg/ml albumin.

Tube 1: incubation with Concanavalin A (as a positive control experiment)
Tube 2: untreated (as a negative control experiment, background)
Tube 3: incubated with human albumin
Tube 4: incubated with human β-actin
Tube 5: incubated with human GAPDH
Tube 6: incubated with human insulin
Tube 7: untreated (as a negative control experiment, no difference to tube 2).

**Figure 2:**
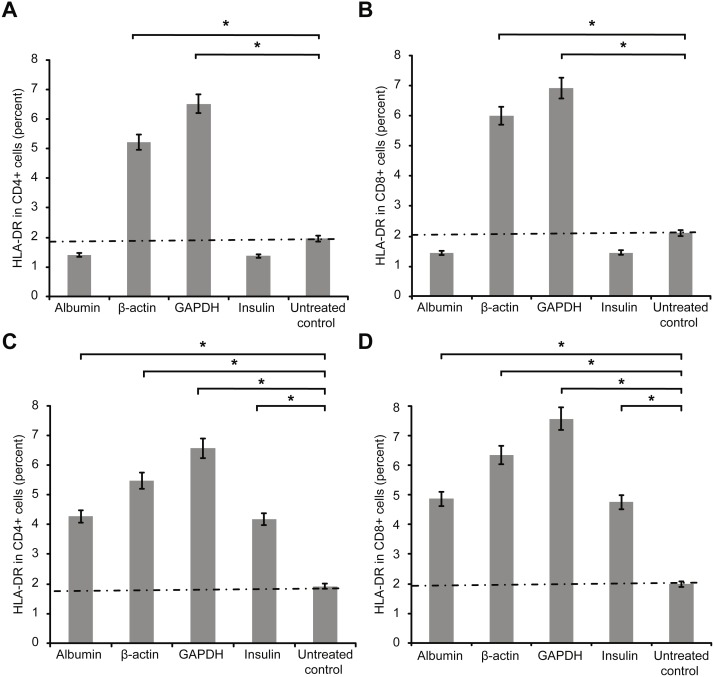
Results of extracellular staining for activated T cell surface marker HLA-DR on CD4+ (A) and CD8+ T cells (B). The percentage of activated cells decreased among both CD4+ and CD8+ T cells incubated overnight with albumin or insulin, indicating their inhibition. In contrast, the percentage of activated cells increased among both CD4+ and CD8+ T cells incubated with an equal concentration of β-actin or GAPDH (*statistically significant difference, *p* < 0.01). Increase in the concentration (2 mg/ml) of the protein (C and D) increased the percentages of activated HLA-DR+ CD4+ and HLA-DR+ CD8+ double-positive T cells.****

Figure 2 presents the second activation marker HLA-DR+ on CD4+ and on CD8+ T cells. Measurements were performed after three days (72 h) of incubation of cultured T cells with antigens as above and additional co-stimulation with CD28.

**Figure 3:**
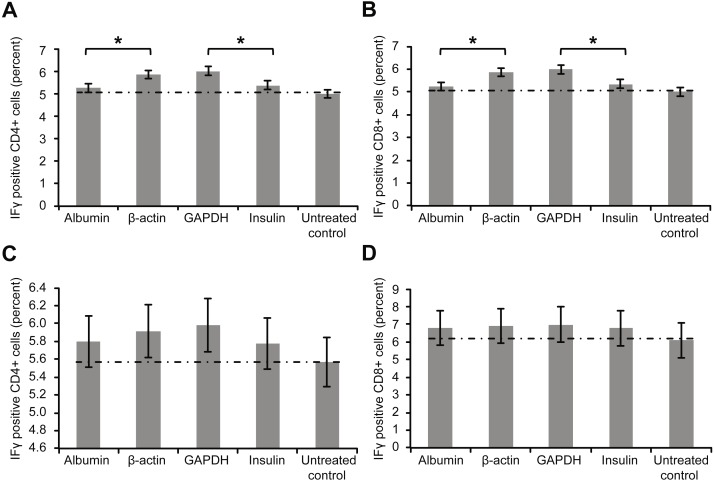
Results of intracellular staining for activation marker IFN-γ in CD69+ CD4+. Results of intracellular staining for activation marker IFN-γ in CD69+ CD4+ (A) and CD69+ CD8+ T cells (B) incubated with different proteins. CD4+ and CD8+ T cells incubated with β-actin or GAPDH showed significantly stronger activation than CD4+ and CD8+ T cells incubated with albumin or insulin. Increase in the concentration (2 mg/ml) of albumin or insulin (C and D) increased the percentage of cytokine-positive T cells (*statistically significant difference, *p* < 0.01).

Figure 3 depicts the intracellular staining results for the activation marker IFγ after whole-blood samples were incubated with the aforementioned proteins. Similar to the trend for surface markers, the levels of intracellular IFγ indicated that incubation with β-actin or GAPDH led to significantly stronger T cell activation than incubation with albumin or insulin. Again, however, incubating the samples with a high concentration of albumin or insulin (2 mg/ml) increased the percentage of cytokine-positive T cells (Figs. 3C and 3D).

**Figure 4:**
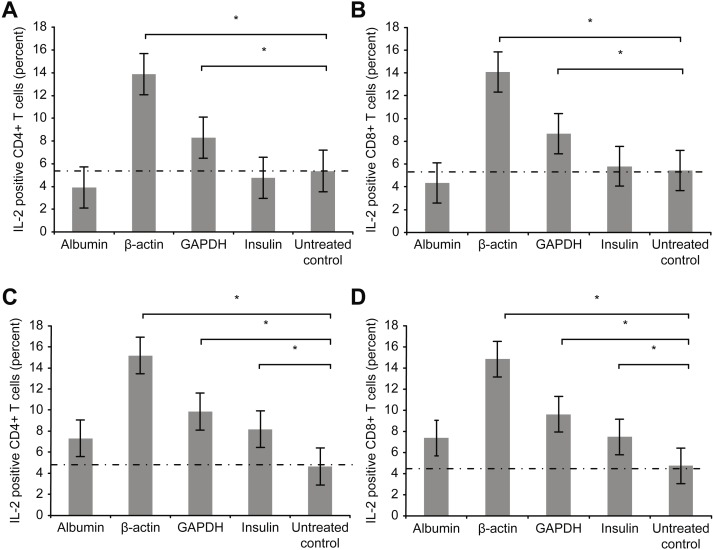
Results of intracellular staining for activation marker IL-2 in CD69+ CD4+. Results of intracellular staining for activation marker IL-2 in CD69+ CD4+ (A) and CD69+ CD8+ T cells (B) incubated with different proteins. CD4+ and CD8+ T cells incubated with β-actin or GAPDH showed significantly stronger activation than CD4+ and CD8+ T cells incubated with albumin or insulin. Increase in the concentration (2 mg/ml) of albumin or insulin (C and D) increased the percentage of cytokine-positive T cells (*statistically significant difference, *p* < 0.01).

Figure 4 depicts the intracellular staining results for the intracellular activation marker interleukin-2 (IL-2) after T cell culture incubation with the aforementioned proteins for 72 h (3 days).

**Figure 5:**
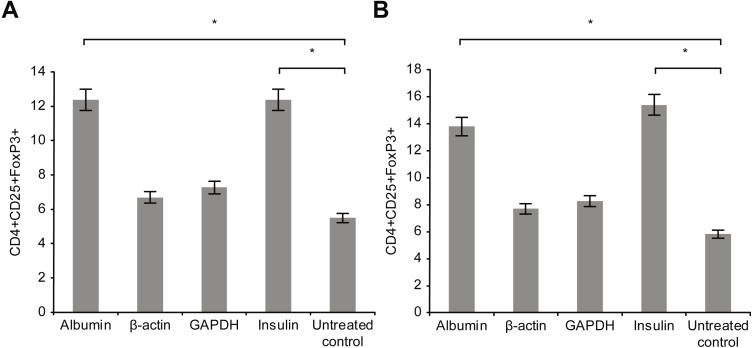
Measurement of CD4+, CD25+, and Fox-P3+ T-reg cells by performing staining. The percentage of T-reg cells increased significantly after incubation with albumin or insulin. However, no significant increase in the percentage of T-reg cells was observed after incubation with β-actin or GAPDH (A). Increase in the concentration (2 mg/ml) of albumin or insulin increased the percentage of T-reg cells compared with that observed after decreasing the concentration of albumin or insulin (*statistically significant difference, *p* < 0.01) (B).

Figure 5 presents the results of T-reg cell measurements performed by staining for CD4+, CD25 +, and FoxP3. The percentage of T-reg cells increased significantly following overnight incubation with albumin or insulin, whereas no significant increase was detected following overnight incubation with β-actin or GAPDH. Therefore, the percentage of T-reg cells and activated T cells, as measured by the expression of surface and intracellular markers, showed differential responses to the four proteins: albumin and insulin increased the percentage of T-reg cells but not activated T cells, whereas β-actin and GAPDH increased the percentage of activated T cells but not the percentage of T-reg cells. Incubating whole blood with a high concentration of albumin or insulin (2 mg/ml) resulted in almost the same percentage of T-reg cells as observed when the cells were incubated with a low concentration (2 µg/ml) of these proteins.

**Figure 6:**
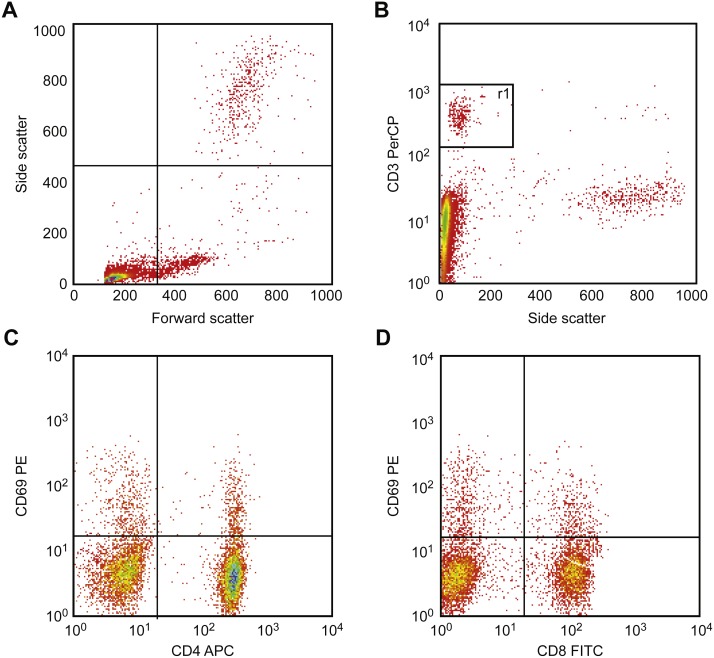
Results of flow cytometric gating for detecting CD69 expression. First, T cells were identified and gated using the CD3 vs. side scatter diagram (A, B). Next, to detect CD69 expression, CD69 was drawn vs. CD4 (C) and vs. CD8 (D) in two separate diagrams, respectively. In these diagrams, double-positive cells (CD69+ CD4+ and/or CD69 + CD8+) were detected and counted. Activation is presented as a percent of double-positive cells.

Figure 6 illustrates the gating strategy. First, T cells are identified and gated using the CD3 vs. side scatter diagram, and then activation was detected. This procedure was performed by drawing CD69 vs. CD4 and vs. CD8 using two separate diagrams. Using these diagrams, activated cells were identified as double-positive T cells (CD69+ CD4+ and/or CD69+ CD8+).

**Figure 7:**
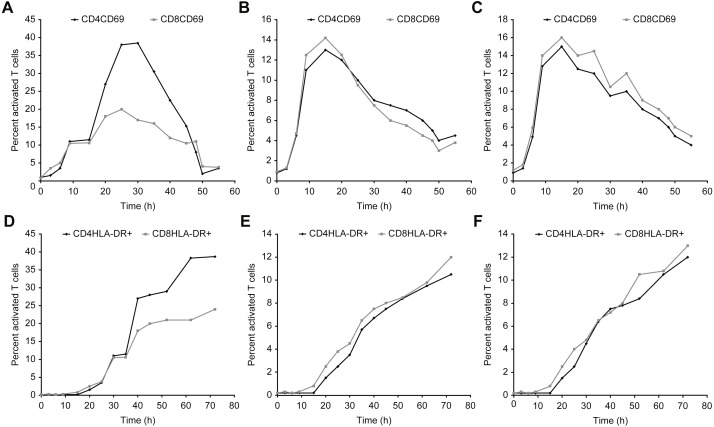
Kinetics of CD69 and HLA-DR expression in T cells after incubation with concanavalin A, β-actin, and/or GAPDH. (A–C) show the CD69 activation curves for concanavalin (A), β-actin (B), and/or GAPDH (C). (D–F) show the HLA-DR activation for concanavalin (D), β-actin (E), and/or GAPDH (F).

**Figure 8:**
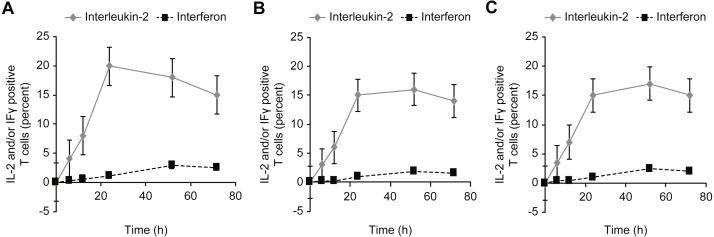
Kinetics of IL-2 and IFN-*γ* expression in T cells incubated with concanavalin A (A), β-actin (B), and/or GAPDH (C).

**Figure 9:**
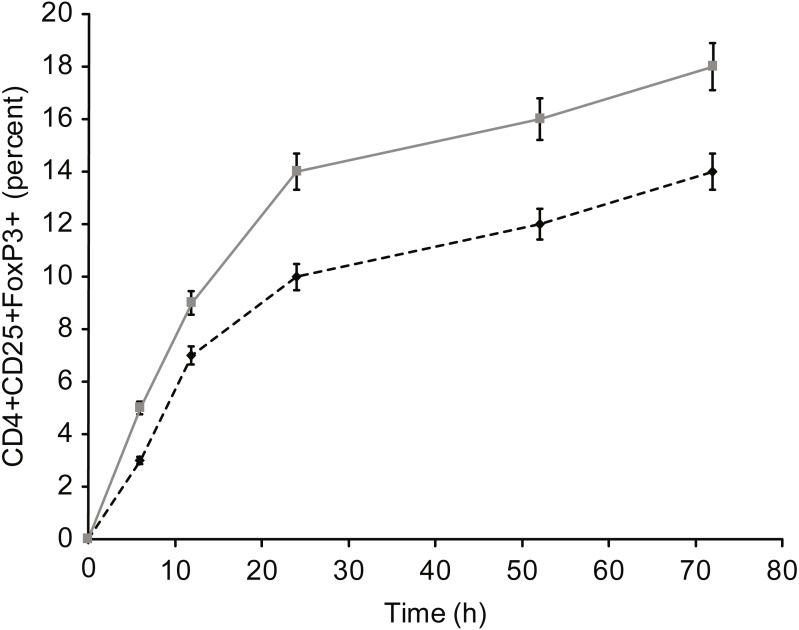
Kinetics of Fox-P3 expression in T cells incubated with insulin.

Figures 7–9 show the kinetics of the cell activations and/or of the cell differentiation.

Table 1 shows the expression-level of markers and numbers of T cells and Tregs before incubation period (expression level at time-point zero).

**Table 1:** Expression-level of markers and cytokines and numbers of T cells and Tregs before incubation period. Expression level at time-point zero.

Marker	Expression-Level in number of positive cells/µl
CD3+	6,570/ µl
CD4+	453/µl
CD8+	319/µl
CD4+/CD8+	1.44
CD4+ CD69+	24/µl
CD8+ CD69+	15/µl
CD3+ HLADR+	53/µl
CD4+ HLADR+	33/µl
CD8+ HLADR+	20/µl
Treg	75/µl

An intracellular cytokine assay (FastImmune) was used to determine the downstream effects of exposure to intracellular proteins by examining the effects on intracellular cytokine production after incubating human whole blood with human proteins (Figs. 3A and 3B). In addition, the expressions of IFN-γ and IL-2 as activation markers of the corresponding T cells were confirmed.

T cell activation was significantly stronger when whole blood was incubated with the intracellular protein β-actin or GAPDH than when incubated with extracellular albumin or insulin. Under physiological conditions, extracellular proteins are normally present in the extracellular space and thus are generally ignored by the immune system. Therefore, incubation with albumin or insulin leads to a much weaker activation than incubation with β-actin or GAPDH.

Incubation with high concentrations of albumin or insulin resulted in high intracellular concentrations of the proteins, leading to an equal level of activation compared with β-actin or GAPDH. This activation was observed for CD69+ T cells, HLA-DR + T cells, and cytokine-positive T cells (IFg and IL-2). In addition, the influence of these proteins on the formation of T-reg cells (Fig. 3) was investigated, and a trend opposite to that of CD4+ and/or CD8 + effector T cells was observed. In particular, T-reg cells were strongly induced by albumin or insulin, which explains why exposure to albumin or insulin (extracellular proteins) suppressed CD4+ and CD8+ T cell activation: incubation with albumin or insulin suppressed T cell activation via the formation of T-reg cells.

The addition of a small amount of a human protein resulted in increased extracellular concentrations of that protein, and T cell activation was inhibited by the induction of T-reg cells after exposure to albumin or insulin (extracellular proteins). In addition, incubation with a high concentration of albumin or insulin led to significant induction of T-reg cells nearly equivalent to that following exposure to a low concentration. Conversely, when GAPDH or β-actin (both intracellular proteins) was extracellularly added at both high and low concentrations, T cells recognized these proteins as non-self-molecules, resulting in both activation at the T cell surface (CD69 and HLA-DR) and cytokine expression (IFγ and IL-2).

## Discussion

Both surface marker staining for CD4+ and CD8+ T lymphocyte activation (CD69+ and HLA-DR+) and intracellular cytokine detection (IFg and IL-2) showed that pre-incubation with albumin or insulin resulted in a low percentage of activated T lymphocytes. However, contrasting results were obtained when the T cells were pre-incubated with human GAPDH or β-actin. Pre-treatment with these proteins resulted in a marked, statistically significant increase in the percentage of activated T lymphocytes, as assessed by the percentages of lymphocytes positive for CD69 (extracellular activation marker) CD4+ and/or CD8+ T lymphocytes, HLA-DR positive CD4+ and/or CD8 + lymphocytes, and/or T lymphocytes positive for intracellular cytokines (IFγ and/or IL-2).

Albumin and insulin are both physiological serum proteins located in the extracellular space and serum; therefore, they should not be able to provoke an immune response or activate T lymphocytes. The present study hypothesized that albumin and insulin contain immunomodulatory sequences. Indeed, immunomodulatory sequences have already been reported in the Fab and Fc termini of immunoglobulins (T-regitope sequences) (De Groot et al., 2008). These or similar sequences may also be present in other serum proteins, including albumin and/or insulin (De Groot et al., 2008), which would explain the immunomodulatory effects of both these proteins on T cell activation.

In contrast, under physiological conditions, the intracellular cytoplasmic proteins GAPDH and β-actin are not found in the serum nor extracellular space. The results of the present study indicate that these proteins are able to induce the differentiation and activation of human T lymphocytes, specifically CD4+ and CD8+, an observation that was experimentally confirmed at both the surface marker and intracellular cytokine levels. Accordingly, this activation of human CD4+ and CD8+ T lymphocytes should be effective and should promote relevant immune responses.

The findings here clearly demonstrate that some physiological intracellular proteins have the potential to activate relevant percentages of CD4+ and CD8+ T lymphocytes, in turn promoting surface marker and intracellular cytokine expression and subsequent activation of the immune system. In contrast, albumin and insulin, which are both physiological extracellular proteins, do not possess the potential to activate T cells, at least under physiologically low concentrations. However, they can induce the formation of T-reg cells. These findings are consistent with common immunological research findings (De Groot et al., 2008; Kaufmann, 1993) and with the characteristics of the evaluated proteins.

T cell activation is closely related to and necessary for the activation of B cells (Katz & Benacerraf, 1972). Several authors have described the occurrence of autoreactive activated T cells (Pauken et al., 2015; Chiorean, Mahler & Sitaru, 2014; Serre, Fazilleau & Guerder, 2015) in healthy subjects (Pauken et al., 2015) and in diverse diseases (Chiorean, Mahler & Sitaru, 2014) and disease models (Serre, Fazilleau & Guerder, 2015).

Physiologically speaking, extracellular proteins should not lead to the activation of endogenous T cells *in vivo*, and the corresponding sequences of TCRs on CD4+ and CD8+ T cells should be absent due to negative selection. However, although negative selection eliminates autoreactive T cells in the thymus, it does not completely eliminate all autoreactive T cells (Pauken et al., 2015; Chiorean, Mahler & Sitaru, 2014; Serre, Fazilleau & Guerder, 2015). In addition, counter-regulatory mechanisms lead to reduced activation of T lymphocytes when extracellular proteins are added. Alternatively, CD4+ cells may detect the above-mentioned substances, leading directly to reduced activation of both CD4+ and CD8+ T cells. In the present study, results differed when GAPDH and/or β-actin were added: because both these proteins are intracellular, they were able to activate both CD4+ and CD8 + T lymphocytes, even at low concentrations.

The parallel activation of both CD4+ and CD8+ T lymphocytes by GAPDH or β-actin can be explained by the concept of cross-presentation: both intracellular proteins are phagocytosed and presented via MHC-I and MHC-II receptors, which can lead to the activation of both CD4+ and CD8+ T lymphocytes or cross-presentation (Rock, 2006). However, different results were obtained when high concentrations of albumin or insulin were added (Figs. 1C and 1D); namely, a dramatic increase in CD8+ T lymphocyte activation, even though CD4+ cells were not activated or only marginally activated.

The present study had hypothesized that T cell activation by intracellular proteins, including β-actin or GAPDH, would also depend on the amount of protein added and that an increased amount would enhance T cell activation. However, this hypothesis could not be confirmed using β-actin or GAPDH. In fact, reduced T cell activation was observed when cells were incubated with high concentrations of β-actin or GAPDH. This effect may be explained by osmotic cellular gradients.

In addition, this novel interpretation of the development of autoimmune diseases fits well with multiple observations described in the literature. For example, the hypothesis and results of the present study are in line with the recent finding that insulin often acts as an autoantigen in type 1 diabetes (Kent et al., 2005; Nakayama et al., 2005; Von Herrath, 2005; Wilson, 2005). In addition, the present study fits well with the observation that myocardial infarction can be accompanied and complicated by autoimmune reactions (Liao & Cheng, 2006).

Currently, the mosaic of autoimmunity is only partially understood (Gershwin, 2008). However, the expression of autoantibodies, which are often associated with autoimmune disease, increases in patients during early disease stages. Over the past 10 years, such autoantibodies have increasingly been demonstrated to have high diagnostic value (Gershwin, 2008). Notably, many of these autoantibodies are directed against intracellular molecules themselves, including ribonucleoproteins (RNPs) and double-stranded DNA (ds DNA) in systemic lupus patients (SLE-patients) and centromeres and type 1 topoisomerase in patients with scleroderma (Gershwin, 2008). Regardless, the mechanisms underlying this process remain poorly understood. The hypothesis and results of the present study could provide a possible explanation for such nonspecific, bystander-autoimmune phenomena.

Recently, cellular ubiquitination and proteolysis systems have been proposed as being closely correlated with the balance between immunity and tolerance (Gomez-Martin, Diaz-Zamudio & Alcocer-Varela, 2008).

CD4+ T cells are known to play an important role in the development of autoimmune diseases (Slavin et al., 2002), with their inhibition leading to the suppression of disease development (Slavin et al., 2002). Interestingly, transfected CD4+ T lymphocytes are used as the preferred vector in experimental gene therapy to treat autoimmune diseases in animals (Slavin et al., 2002).

Two well-accepted alternative hypotheses for the development of autoimmune diseases are the virus hypothesis and the molecular mimicry hypothesis. The viral hypothesis states that new, unknown viruses are responsible for the development of autoimmune diseases; however, this hypothesis remains unconfirmed in light of current knowledge (Denman & Rager-Zisman, 2004). Accordingly, future studies that attempt to detect unknown viruses should be performed, and the strong postulates of Koch may need to be changed (Denman & Rager-Zisman, 2004).

Cardiovascular disease has been shown to occur much more frequently in patients with rheumatoid arthritis and to more often lead to death in these patients than in healthy individuals (Sarzi-Puttini et al., 2005).

The present study has shown that, in principle, physiological autoreactive T lymphocytes are present even in healthy individuals. Several studies have shown the presence of a large repertoire of T lymphocytes oriented toward various organs and/or tissues in healthy mice (Sharma et al., 2007). If these autoimmune T lymphocytes expand, an autoimmune attack may occur (Sharma et al., 2007). In addition, several authors have reported the spontaneous development of autoimmune diseases in mice; these diseases target the skin, lungs, liver, and tail when T-reg cells (CD4+, CD25+, and Foxp3+ cells) are absent (Foxp3 protein-deficient scurfy mice Sharma et al., 2007; Davies, 2008; Goodnow et al., 2005). These results show that additional mechanisms in healthy individuals prevent autoimmune attacks, including important T-reg cells, which the present study investigated. However, the immune system is regulated by other control systems; indeed, multiple control systems appear to be necessary, particularly due to the possibility of normal, healthy individuals developing autoimmune diseases.

These mechanisms are both important and necessary for the accurate control of the self/non-self recognition process.

Over the past decade, several studies have reported that all immune-system components appear to be involved in the development and modulation of atherosclerosis (Bassi et al., 2009). In particular, pentraxins and antibodies appear to play important roles. Pentraxins are ancient multimer molecules that are also important to the recognition of self- and non-self structures and are functionally complementary to the cellular recognition of self- and non-self molecules (Bottazzi et al., 2006). As described above, these observations match the hypothesis outlined by the present study.

Recently, a mechanism has been described in which intracellular endogenous proteins are presented by MHC-II due to autophagy by antigen-presenting cells (Münz, 2012). This effect might be dependent on the exact physiological context and on the concentration of the respective intracellular protein in the plasma, as shown by the present study. The occurrence of this autophagy-driven presentation of intracellular peptides to MHC-II during infections and similar situations could explain the hygiene hypothesis. To date, autophagy has been described in connection with infection with the herpes simplex virus and influenza viruses (Münz, 2012). If no infection occurs in early childhood, then the MHC repertoires are different, and no cross-presentation occurs. However, if infections occur in early childhood, the intracellular antigen spectra might better display via the MHC-II pathway, and resistance to autoimmune diseases might increase.

Herein a strong division of the antigenic spectrum into an extracellular part, consisting of CD4 T cell receptors, and an intracellular part, consisting of CD8 T cell receptors is postulated.

With regard to the phenomenon of cross-presentation (presentation of exogenous proteins by the MHC-I receptor), the following must be said.

Scientific work in recent years has shown that cross-presentation is a limited, highly specialized, and precisely regulated process (Fehrens et al., 2014). The process is limited only to specific specialized cells (DC) (Fehrens et al., 2014) and specific organelles within these cells (Brode & Macary, 2004). In addition, many specialized enzymes are involved in precisely regulating the process (Brode & Macary, 2004).

Furthermore, as has been described, only antigens already bound to specific receptors (immunoglobulins and Fc receptors) can be taken up by these cells and then processed by cross-presentation (Platzer, Stout & Fiebiger, 2014).

However, if this is the case, then the phenomenon of cross-presentation does not in any way contradict the division of the antigen spectrum into an extracellular and intracellular spectrum, as described here, and does not preclude the representation of these divisions by the various T cell receptors and the various lymphocyte populations (CD4+ and CD8+ T lymphocytes).

On the contrary, if uptake and processing of an antigen is possible via cross-presentation only after binding to specific receptors (immunoglobulins and Fc receptors Platzer, Stout & Fiebiger, 2014), then that antigen has already been recognized as foreign, ensuing that the antigen is not a body-own-specific structure of the extracellular space. This strongly supports the correctness of the relationship postulated here.

In order to establish these findings it is necessary to test a line of further extracellular and intracellular proteins for their immune activation potential in future studies. In addition, species difference studies should also be performed in future research.

## Conclusions

Overall, in addition to the location being important for immunization, as described herein, other mechanisms may be relevant to the development of autoimmune diseases, including molecular mimicry, viral infections, and redox reactions of antibodies, all of which may be responsible for the development of autoantibodies. However, the results of the present study suggest an additional mechanism; namely, the misguided formation of proteins that become antigens.

Several aspects of the experiments conducted in the present study have been reported in part by other researchers, although their research was conducted within different contexts. For instance, the existence of an autoreactive T cell repertoire, even in healthy individuals, has been reported by several authors (Kamate et al., 2007; Walker & Abbas, 2002; Munk et al., 1989; Zou et al., 2008; Vella et al., 2009). Their findings are consistent with those of the present study, and a hypothetical explanation for this phenomenon is provided herein. In addition, an autoreactive T cell response toward insulin was reported by Ito and Yang but only in patients with autoimmune diabetes (Ito et al., 1993; Yang et al., 2014). Lastly, induction of T-reg cells in response to a human protein—namely, intravenous immunoglobulin—was reported by Maddur et al. (2001) and Cousens et al. (2013).

The present study confirmed and extended their experimental results, placed them within the context of disease development, and provided an explanation. The present study is in line with the following novel proposal of autoimmune regulation: When self-molecules occur at the wrong place in an intact organism, they are recognized as non-self-molecules, leading to the activation of immune cells. Therefore, structural protein abnormalities, mutations, or errors, including those in signaling, are not necessary to induce immunization against the organism itself (self-immunization); indeed, a mere mislocalized protein is sufficient.

##  Supplemental Information

10.7717/peerj.4462/supp-1 Data S1 Raw data

10.7717/peerj.4462/supp-2 Data S2 Negative (background) controls and Concanavalin A controls Samples incubated without anything (negative controls, background controls) and samples incubated with Concanavalin A (positive controls).

## Additional Information and Declarations

### Data Availability

The following information was supplied regarding data availability: The raw data are provided in a Supplemental File.

## References

[ref-1] Bassi N, Zampieri S, Ghirardello A, Tonon M, Zen M, Cozzi F, Doria A (2009). Pentraxins, anti-pentraxin antibodies, and atherosclerosis. Clinical Reviews in Allergy & Immunology.

[ref-2] Bottazzi B, Garlanda C, Salvatori G, Jeannin P, Manfredi A, Mantovani A (2006). Pentraxins as a key component of innate immunity. Current Opinion in Immunology.

[ref-3] Brode S, Macary P (2004). Cross-presentation: dendritic cells and macrophages bite off more than they can chew!. Immunology.

[ref-4] Chiorean R, Mahler M, Sitaru C (2014). Molecular diagnosis of autoimmune skin diseases. Romanian Journal of Morphology and Embryology.

[ref-5] Cousens L, Najafian N, Mingozzi F, Elyaman W, Mazer B, Moise L, Messitt T, Su Y, Sayegh M, High K, Khoury S, Scott D, De Groot A (2013). *In vitro* and *in vivo* studies of IgG-derived Treg epitopes (Tregitopes): a promising new tool for tolerance induction and treatment of autoimmunity. Journal of Clinical Immunology.

[ref-6] Davies AJS (2008). Immunological tolerance and the autoimmune response. Autoimmunity Reviews.

[ref-7] De Groot A, Moise L, McMurry J, Wambre E, Van Overtvelt L, Moingeon P, Scott DW, Martin W (2008). Activation of natural regulatory T cells by IgG Fc–derived peptide “T-regitopes”. Blood.

[ref-8] Denman AM, Rager-Zisman B (2004). Viruses and autoimmune diseases—adapting Koch’s postulates. Autoimmun Rev.

[ref-9] Fehrens C, Unger W, Garcia-Vallejo J, Van Kooyk Y (2014). Understanding the biology of antigen cross-presentation for the design of vaccines against cancer. Frontiers in Immunology.

[ref-10] Gershwin ME (2008). Editorial. The mosaic of autoimmunity. Autoimmun Rev.

[ref-11] Gomez-Martin D, Diaz-Zamudio M, Alcocer-Varela J (2008). Ubiquitination system and autoimmunity: the bridge towards the modulation of the immune response. Autoimm Rev.

[ref-12] Goodnow CC, Sprent J, Fazekas de St Groth B, Vinuesa CG (2005). Cellular and genetic mechanisms of self-tolerance and autoimmunity. Nature.

[ref-13] Ito Y, Nieda M, Uchigata Y, Nishimura M, Tokunaga K, Kuwata S, Obata F, Tadokoro K, Hirata Y, Omori Y (1993). Recognition of human insulin in the context of HLA-DRB1*0406 products by T cells of insulin autoimmune syndrome patients and healthy donors. Journal of Immunology.

[ref-14] Kamate C, Lenting P, Van den Berg H, Mutis T (2007). Depletion of CD4+/CD25 high regulatory T cells may enhance or uncover factor VIII-specific T-cell responses in healthy individuals. Journal of Thrombosis and Haemostasis.

[ref-15] Katz DH, Benacerraf B (1972). The regulatory influence of activated T cells on B cell responses to antigen. Advances in Immunology.

[ref-16] Kaufmann S (1993). Immunity to intracellular bacteria. Annual Review of Immunology.

[ref-17] Kent SC, Chen Y, Bregoli L, Clemmings SM, Kenyon NS, Ricordi C, Hering BJ, Hafler DA (2005). Expanded T-cells from pancreatic lymph nodes of type 1 diabetic subjects recognize an insulin epitope. Nature.

[ref-18] Liao YH, Cheng X (2006). Autoimmunity in myocardial infarction. International Journal of Cardiology.

[ref-19] Maddur MS, Vani J, Hegde P, Lacroix-Desmazes S, Kaveri SV, Bayry J (2001). Inhibition of differentiation, amplification, and function of human TH17 cells by intravenous immunoglobulin. Journal of Allergy and Clinical Immunology.

[ref-20] Munk M, Schoel B, Modrow S, Karr R, Young R, Kaufmann S (1989). T lymphocytes from healthy individuals with specificity to self-epitopes shared by the mycobacterial and human 65-kilodalton heat shock protein. Journal of Immunology.

[ref-21] Münz (2012). Antigen processing for MHC class II presentation via autophagy. Frontiers in Immunology.

[ref-22] Nakayama M, Abiru N, Moriyama H, Babaya N, Liu E, Miao D, Yu L, Wegmann DR, Hutton JC, Elliott JF, Eisenbarth GS (2005). Prime role for an insulin epitope in the development of type 1 diabetes in NOD mice. Nature.

[ref-23] Pauken K, Nelson C, Martinov T, Spanier J, Heffernan J, Sahli N, Quarnstrom C, Osum K, Schenkel J, Jenkins M, Blazar B, Vezys V, Fife B (2015). Cutting edge: identification of autoreactive CD4+ and CD8+ T cell subsets resistant to PD-1 pathway blockade. Journal of Immunology.

[ref-24] Platzer B, Stout M, Fiebiger E (2014). Antigen cross-presentation of immune complexes. Frontiers in Immunology.

[ref-25] Rock LK (2006). Exiting the outside world for cross-presentation. Immunity.

[ref-26] Sarzi-Puttini P, Atzeni F, Shoenfeld Y, Ferraccioli G (2005). TNFa, rheumatoid arthritis, and heart failure: a rheumatological dilemma. Autoimmun Rev.

[ref-27] Serre L, Fazilleau N, Guerder S (2015). Central tolerance spares the private high-avidity CD4+ T-cell repertoire specific for an islet antigen in NOD mice. European Journal of Immunology.

[ref-28] Shah DK, Zúñiga-Pflücker JC (2014). An overview of theintrathymic intricacies of T cell development. Journal of Immunology.

[ref-29] Sharma R, Jarjour WN, Zheng L, Gaskin F, Fu SM, Ju ST (2007). Large functional repertoire of regulatory T-cell suppressible autoimmune T cells in scurfy mice. Journal of Autoimmunity.

[ref-30] Slavin AJ, Tarner IH, Nakajima A, Urbanek-Ruiz I, McBride J, Contag CH, Fathman CG (2002). Adoptive cellular gene therapy of autoimmune disease. Autoimmun Rev.

[ref-31] Vella L, Yu M, Fuhrmann S, El-Amine M, Epperson D, Finn O (2009). Healthy individuals have T-cell and antibody responses to the tumor antigen cyclin B1 that when elicited in mice protect from cancer. Proceedings of the National Academy of Sciences of the United States of America.

[ref-32] Von Herrath M (2005). Immunology: insulin trigger for diabetes. Nature.

[ref-33] Walker L, Abbas A (2002). The enemy within: keeping self-reactive T cells at bay in the periphery. Nature Reviews Immunology.

[ref-34] Wilson DB (2005). Immunology: insulin auto-antigenicity in type 1 diabetes. Nature.

[ref-35] Yang J, Chow I-T, Sosinowski T, Torres-Chinn N, Greenbaum C, James E, Kappler J, Davidson H, Kwoka W (2014). Autoreactive T cells specific for insulin B:11-23 recognize a low-affinity peptide register in human subjects with autoimmune diabetes. Proceedings of the National Academy of Sciences of the United States of America.

[ref-36] Zinkernagel RM (2003). On natural and artificial vaccinations. Annual Review of Immunology.

[ref-37] Zinkernagel RM, Bachmann MF, Kuendig TM, Oehen S, Pircher HP, Hengartner H (1996). On immunological memory. Annual Review of Immunology.

[ref-38] Zinkernagel RM, Hengartner H (2006). Protective immunity by pre-existent neutralizing antibody titers and preactivated T cells but not by so-called immunological memory. Immunological Reviews.

[ref-39] Zou J, Hannier S, Cairns L, Barker R, Rees A, Turner A, Phelps R (2008). Healthy individuals have good pasture autoantigen-reactive T cells. Journal of the American Society of Nephrology.

